# Monophosphoryl lipid A ameliorates radiation-induced lung injury by promoting the polarization of macrophages to the M1 phenotype

**DOI:** 10.1186/s12967-022-03804-x

**Published:** 2022-12-14

**Authors:** Xingdong Guo, Lehui Du, Na Ma, Pei Zhang, Yuan Wang, Yanan Han, Xiang Huang, Qian Zhang, Xin Tan, Xiao Lei, Baolin Qu

**Affiliations:** 1 Department of Radiation Oncology, Chinese PLA General Hospital, Beijing, China; 2 Medical School of Chinese PLA, Beijing, China; 3 School of Biological Science and Medical Engineering, Beihang University, Beijing, China

**Keywords:** Monophosphoryl lipid A, Radiation-induced lung injury, Radioprotection, Macrophages, Exosomes, Epithelial-mesenchymal transition

## Abstract

**Background:**

Radiation-induced lung injury (RILI) often occurs during clinical chest radiotherapy and acute irradiation from accidental nuclear leakage. This study explored the role of monophosphoryl lipid A (MPLA) in RILI.

**Materials and Methods:**

The entire thoracic cavity of C57BL/6N mice was irradiated at 20 Gy with or without pre-intragastric administration of MPLA. HE staining, Masson trichrome staining, and TUNEL assay were used to assess lung tissue injury after treatment. The effect of irradiation on the proliferation of MLE-12 cells was analyzed using the Clonogenic assay. The effect of MPLA on the apoptosis of MLE-12 cells was analyzed using flow cytometry. Expression of γ-H2AX and epithelial-mesenchymal transition (EMT) markers in MLE-12 cells was detected by immunofluorescence and Western blot, respectively.

**Results:**

MPLA attenuated early pneumonitis and late pulmonary fibrosis after thoracic irradiation and reversed radiation-induced EMT in C57 mice. MPLA further promoted proliferation and inhibited apoptosis of irradiated MLE-12 cells in vitro. Mechanistically, the radioprotective effect of MPLA was mediated by exosomes secreted by stimulated macrophages. Macrophage-derived exosomes modulated DNA damage in MLE-12 cells after irradiation. MPLA promoted the polarization of RAW 264.7 cells to the M1 phenotype. The exosomes secreted by M1 macrophages suppressed EMT in MLE-12 cells after irradiation.

**Conclusion:**

MPLA is a novel treatment strategy for RILI. Exosomes derived from macrophages are key to the radioprotective role of MPLA in RILI.

## Introduction

Radiation-induced lung injury (RILI) is one of the most common complications caused by thoracic radiotherapy [1]. Patients receiving radiation therapy, workers in nuclear power plants, or people exposed to high radiation levels as a result of a nuclear accident are at risk of RILI [2]. RILI, accompanied by radiation pneumonitis (RP) and radiation fibrosis, occurs in approximately 5–20% of patients with thoracic radiotherapy, limiting the use of maximum irradiation dose. This results in poor tumor treatment control and may lead to dyspnea, lowering the quality of life of lung cancer patients [3]. RILI can activate the production of various cytokines, infiltration of inflammatory cells, fibroblasts, and tissue remodeling, ultimately impairing lung function and causing respiratory failure [4]. So far, amifostine is the only drug approved by FDA for radioprotection. However, due to its adverse side effects, such as vomiting, nausea and hypotension, it is not routinely used clinically. Therefore, the development of new therapeutic drugs is of great significance.

Toll-like receptors (TLRs) are type I transmembrane proteins that mediate the recognition of pathogen-associated molecular patterns (PAMPs). So far, 10 and 12 functional TLRs have been found in humans and mice, respectively [5]. TLRs are essential human innate immunity receptors that participate in certain immune responses and immune-related disorders. Irradiation (IR) strongly activates TLRs in human cells [6]. In recent years, numerous studies have demonstrated the protective effects of TLRs against IR. Kutikhin et al. [7] revealed that TLRs (TLR4, TLR7, TLR8, and TLR9) participate in DNA repair. Burdelya et al. [8] demonstrated that CBLB502, a TLR5 activation agonist, alleviates acute radiation syndrome and improves the survival of mice after radiation exposure. Collectively, the reports indicated that TLRs play essential radiative roles.

Monophosphoryl lipid A (MPLA) is a TLR4 agonist that is 10,000 times less toxic than Lipopolysaccharide (LPS) [9, 10]. Guo et al. [11] demonstrated that MPLA protects the intestines against ionizing radiation damage by activating the TLR4 signaling pathway. A related study [12] revealed that macrophage-derived exosomes stimulated by MPLA played an essential role in protecting the testis against radiation. Epithelial-mesenchymal transition (EMT) plays a vital role in RILI [13]. Polydatin alleviates RILI by inhibiting EMT [14]. Reversing EMT may alleviate RILI. In the present study, we demonstrated that MPLA effectively ameliorated radiation injury and inflammation in lung tissues. MPLA reverses EMT via macrophage-derived exosomes. Thus, MPLA is a promising option for treating RILI.

## Materials and methods

### Irradiation of animals

Adult male C57BL/6N mice (6–8-week-old, 18‐20 g) were purchased from Vital River Experimental Animal Company (Beijing, China). The protocols for animal experiments were approved by the Animal Care and Use Committee of the Academy of Military Medical Sciences (Beijing, China). The mice were maintained in rooms under a 12-h light/dark cycle, 20–26 °C, and at a relative humidity of 40–70%. The mice were acclimatized for one week and were fed enough food and water before the experiments.

Before irradiation, the mice were randomly divided into four groups as follows: normal control (CON group); MPLA without irradiation group (MPLA group); irradiation only group (IR group), and irradiation plus MPLA group (MPLA + IR group). MPLA was purchased from InvivoGen (Lot: 5978–42-01). Based on our previous research results[12], mice in the MPLA + IR group and MPLA group were injected intragastrically with 50 μg/kg MPLA dissolved in 0.1 mL phosphate buffer saline 12 h before irradiation. Mice in the IR group were injected with 0.1 mL phosphate buffer saline. In the previous research, the RILI model has been successfully constructed and verified many times [15]. Mice were then anesthetized by intraperitoneal injection with 1% pentobarbital sodium (50 mg/kg body weight) before thoracic irradiation (20 Gy) with a ^60^Co γ-ray (Beijing Institute of Radiation) at a rate of 60.87 cGy/min. Except for the thorax, the other parts of the mice were covered with 10-cm-thick lead bricks.Lung tissue samples were collected at weeks 1, 4, 8, and 16 after radiation.

### Cell culture and treatment

The mice alveolar epithelial cell (MLE‐12), the mice monocyte cell line (RAW 264.7) and the human lung epithelial cell (Beas-2B) were obtained from Otwo Biotech (Shenzhen, China). The cells were cultured in DMEM medium (10% fetal calf serum) at 37 °C in a 5% CO_2_ humidified chamber. MLE-12 cells were radiated at dose of 8 Gy (clonogenic assay with 0 Gy, 2 Gy, 4 Gy, and 8 Gy) [16].

### Conditioned culture

We used different culture supernatants as a conditioned medium to observe their effects on MLE-12 cells. We cultured the RAW 264.7 cells when the confluence was approximately 50–60%, replaced the original medium with DMEM medium, and continued to cultivate for 12 h. After this, we collected the supernatants and centrifuged them for 10 min at 2000 rpm, collected the supernatant as RAWsup; We added 1 µg/mL MPLA to the medium DMEM medium, then maintained cultured RAW264.7 cells for an additional 12 h. Next, collected the supernatants and centrifuged them for 10 min at 2000 rpm, the supernatant was MPLAsup; Unlike MPLAsup, we cultured RAW264.7 for 2 h with a medium containing GW4689 of 20 μM concentration before using MPLA [17, 18], the following steps were the same as the MPLAsup, we collected the supernatant as (GW4869 + MPLA)sup.

### Clonogenic assay

The cell proliferation potential was assessed based on the survival rate of the clones. Specific number of cells was seeded in 6-well plates and irradiated with 0 Gy, 2 Gy, 4 Gy, and 8 Gy. After 7–10 days of incubation, the plates were fixed with paraformaldehyde, stained with 1% crystal violet for 30 min, washed with PBS, and colonies (≥ 50 cells as a colony) counted under a dissecting microscope. The surviving fraction (SF) curve was based on the multi-target single-hit model.

### Apoptosis analysis

FITC Annexin V Apoptosis Detection Kit I (BD Pharmingen) was used for analysising cell appotosis. After different experimental conditions, the MLE-12 cells were washed twice with cold PBS and resuspended in 1X Binding Buffer at 1 × 10^6^ cells/mL. Thereafter, 100 μL of the solution (1 × 10^5^ cells) was transferred into a 5 mL culture tube before adding 5 μL of FITC Annexin V and 5 μL of PI. The cells were gently vortexed and incubated for 15 min at 25 °C in the dark. Then, 400 μL of 1X Binding Buffer was added to each tube. Apoptosis rate analysis was performed within 1 h using flow cytometry.

### Western blot analysis

Protein extracts were prepared from a different group of MLE-12 cells. The samples were incubated for 2 h with primary antibodies against TLR4 (proteintech, 1:5000), γ-H2AX (Cell Signaling Technology; 1:1000), E-cadherin (Cell Signaling Technology, 1:1000), Vimentin (Cell Signaling Technology, 1:1000), and α-SMA (Cell Signaling Technology, 1:1000) in 5% nonfat milk. The samples were then incubated with HRP-conjugated IgG (proteintech, 1:5000) for 1 h.

### Immunofluorescence staining

Immunofluorescence assay was used to detect the number of γ-H2AX foci. Cells were seeded in 24-well plates. After washing with PBS, cells were fixed in 3% paraformaldehyde and permeabilized in 0.1% Triton X-100 in PBS. The cells were then stained with γ-H2AX primary antibody (Cell Signaling Technology; 1:200) and thereafter the secondary antibody (fluorescein goat anti-rabbit IgG, Invitrogen; 1:1000). Cells were stained with a DAPI (Antifade Mounting Medium with DAPI, Beyotime). The images were captured using an Olympus BX60 fluorescent microscope (Olympus America Inc).

### Enzyme-linked immunosorbent assay

The serum levels of tumor necrosis factor-alpha (TNF-α), transforming growth factor-β (TGF-β), IL-1β, and IL-10 were measured using ELISA, following the manufacturer’s instructions (BOSTER).

### TLR4 expression and GO analysis

Data for TLR4 expression were downloaded from the TCGA and GTEx databases (http://gepia2.cancer-pku.cn/#index). GO analysis was analyzed with the R software.

### Statistical analysis

All quantitative data were presented as the mean ± standard error of the mean (SEM). All experiments were performed in 3 independent replicates. Differences between two groups were analyzed using the t-test, while differences among multiple groups were analyzed using the one-way ANOVA and Tukey’s test. Statistical significance was set at 5%. All statistical analyses were two-sided, and data were analyzed using the Prism 9.0 software (GraphPad).

## Results

### MPLA alleviated IR-induced lung injury in mice

**Fig. 1:**
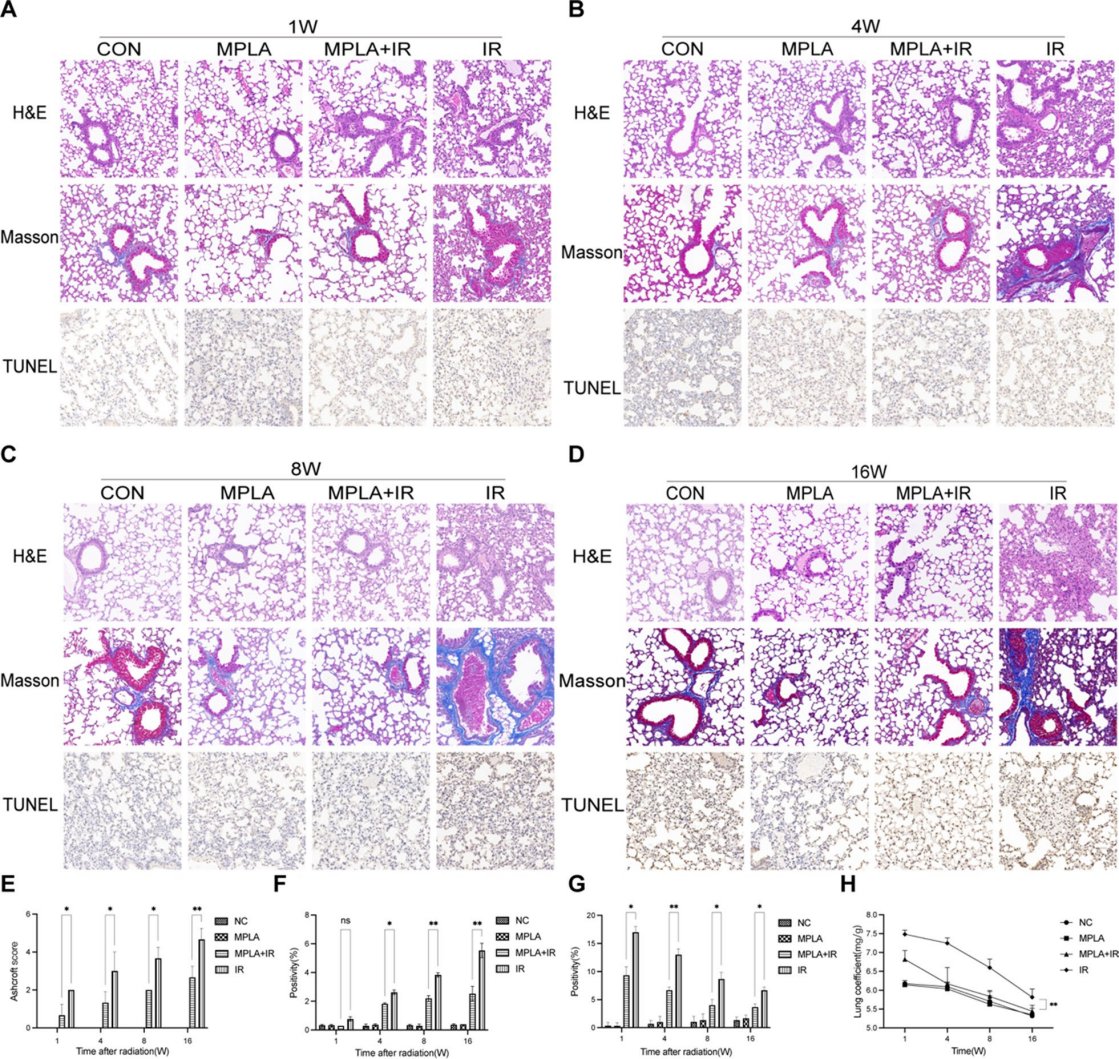
MPLA attenuated irradiation-induced inflammation and structural damage in mice lung tissue. **A**–**D**, Representative images showing the HE staining, Masson trichrome staining and TUNEL assay results of lung tissue sections at 1-week, 4-week, 8-week and 16-week post-irradiation. **E**, Ashcroft scoring of HE staining of lung tissues (The score of NC group and MPLA group was 0 score, one image per mice). **F**, Ashcroft scoring of Masson trichrome staining of lung tissues (one image per mice). **G**, Representative images of the TUNEL assay of lung tissues (one image per mice). **H**, The lung coefficient of mice in each group. Data are shown as the means ± SEM, n = 3 mice per group. *P < 0.05, **P < 0.01, *** P < 0.001,****P < 0.0001

MPLA plays a critical radioprotective role in many tissues except the lung. To investigate the radioprotective effect of MPLA on mice lung tissue, the thoracic regions of mice were irradiated and analyzed. After 20 Gy local irradiation, the infiltration of cells was observed at weeks 1 and 4, and alveolar septal thickening was observed primarily at weeks 8 and 16 by Hematoxylin staining. Compared with the single irradiation group, radiation-induced inflammation and alveolar wall thickening were significantly lower in the MPLA + IR group (Fig. 1A–D). Quantitative analysis showed that MPLA modulated radiation-induced pneumonitis (Fig. 1E).

Advanced pulmonary RILI is mainly characterized by excess fibrosis [19]. Masson’s trichrome staining revealed that collagen deposition increased from 1-week to 16-week post-irradiation. Compared with the single irradiation group, collagen deposition induced by radiation in the MPLA + IR group was significantly low (Fig. 1A–D). Quantitative analysis showed that MPLA inhibited radiation-induced collagen deposition and pulmonary fibrosis (Fig. 1F).

TUNEL assay is a critical method for quantifying IR-Induced apoptosis in mice lungs. The apoptosis rate was analyzed using respective markers. The results showed that the MPLA significantly reduced the apoptosis of alveolar epithelial cells after radiation (Fig. 1A–D). The quantitative analysis presented as Fig. 1G.

Lung coefficient (lung weight/body weight) indicates the degree of pulmonary edema and high vascular permeability. We found that MPLA treatment restored normal lung weight after irradiation (Fig. 1H), suggesting that MPLA prevents RILI in vivo.

### MPLA reversed radiation-induced EMT in mice

**Fig. 2:**
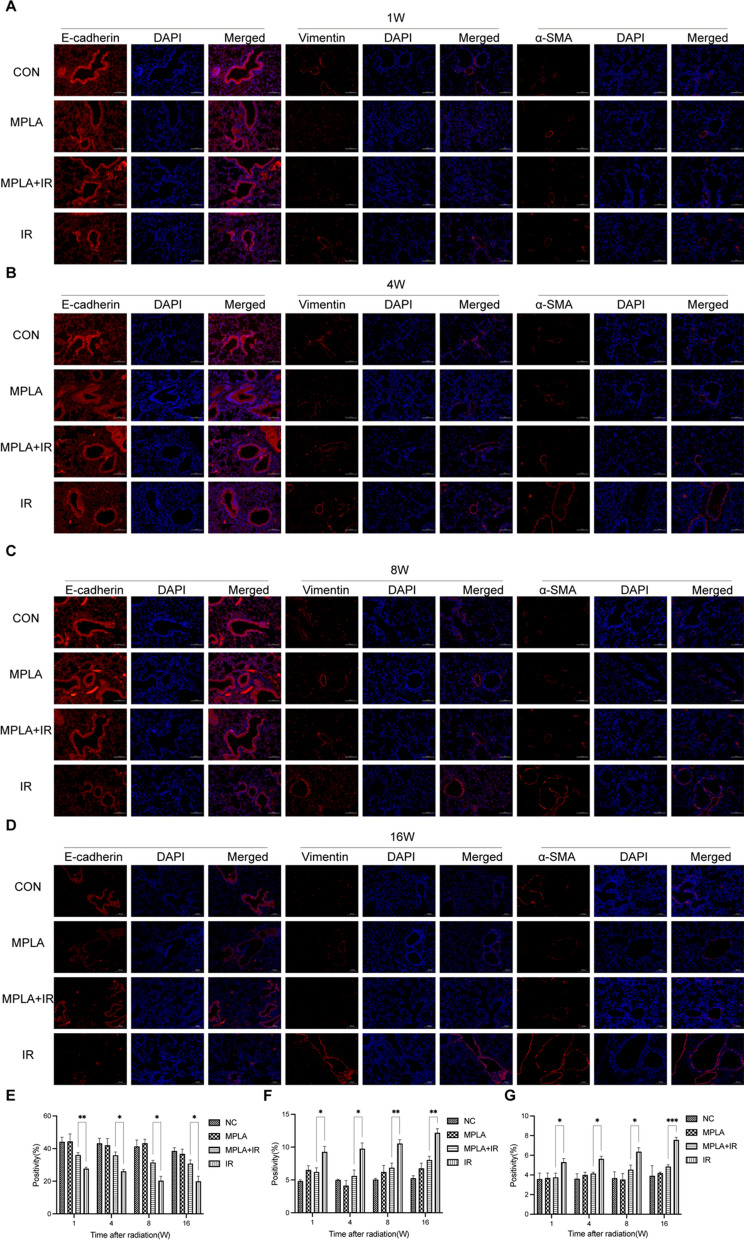
MPLA inhibited radiation-induced EMT in lung tissues. **A**, Representative images showing the staining intensity of E-cadherin, Vimentin, and α-SMA in lung tissue sections at 1-week post-irradiation. **B**, Representative images displaying the staining intensity of E-cadherin, Vimentin, and α-SMA in lung tissue sections at 4-week post-irradiation. **C**, Representative images showing the staining intensity of E-cadherin, Vimentin, and α-SMA in lung tissue sections at 8-week post-irradiation. **D**, Representative images displaying the staining intensity of E-cadherin, Vimentin, and α-SMA in lung tissue sections at 16-week post-irradiation. **E**, The quantification analysis of E-cadherin-positive cells in lung tissues (one image per mice). **F**, The quantification analysis of Vimentin-positive cells in lung tissues (one image per mice). **G**, The quantification analysis of α- SMA positive cells in lung tissues (one image per mice). Data are shown as the means ± SEM, n = 3 mice per group. *P < 0.05, **P < 0.01, *** P < 0.001,****P < 0.0001

There is a close relationship between radiation-induced EMT and pulmonary fibrosis. The expression of EMT markers reflects the EMT severity [20, 21]. In the present study, the expression of EMT markers (E-cadherin and vimentin and α-SMA) in the epithelial lung cells were analyzed by immunofluorescence staining. The results showed that E-cadherin expression was downregulated in the IR group. In contrast, vimentin and α-SMA expression were upregulated in the alveolar epithelium after irradiation. However, MPLA inhibited the downregulated E-cadherin expression induced by radiation and inhibited the overexpression of α-SMA and vimentin in (Fig. 2A–D). Quantitative analysis revealed that irradiation reduced the expression of E-cadherin, but MPLA treatment reversed this phenomenon (Fig. 2E). MPLA inhibited the expression of vimentin and α-SMA in irradiated lung tissues (Fig. 2F–G). This result indicated that MPLA ameliorated radiation-induced EMT in mice lung tissue.

### MPLA promoted the proliferation of MLE-12 cells

**Fig. 3:**
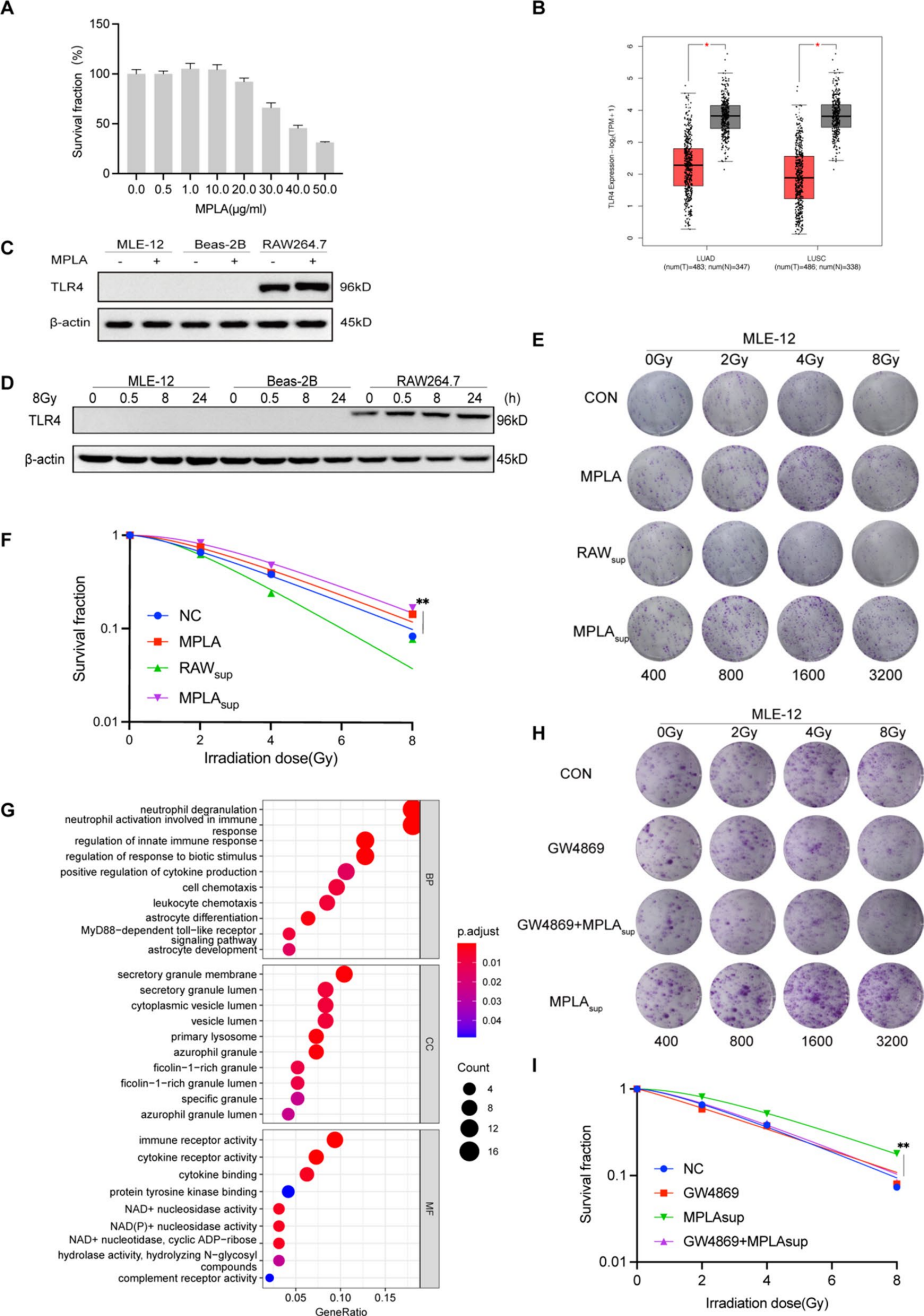
MPLA promoted the proliferation of MLE-12 cells by activating TLR4 on macrophages to trigger the production of exosomes. **A**, Survival fraction of MLE-12 cell at different MPLA concentrations. **B**, The level of TLR4 in tissues from various lung cancer patients and normal tissues. Data were obtained from the TCGA and GTEx databases. **C**, The TLR4 expression detected after MPLA treatment in MLE-12 cells, Beas-2B cells and RAW 264.7 cells. **D**, Protein levels of TLR4 as determined by western analysis in MLE-12 cells, Beas-2B cells and RAW 264.7 cells at different times after irradiation. **E**, **H**, The colony-forming ability of MLE-12 cells exposed to different IR radiations. **F**, **I**, The “multi-target-single-hit-model” for determining the cell viability of MLE-12 cells. G, Top 10 enriched biological processes, molecular functions, cellular components of TLR4 in data obtained from the TCGA database. *P < 0.05, **P < 0.01, *** P < 0.001,****P < 0.0001

Our experiments demonstrated the radioprotective effect of MPLA on mice lung tissue in vivo. In vitro experiments were performed to explore whether MPLA also has a radioprotective effect on mice lung epithelial cells. We tested the toxicity of MPLA on MLE-12 cells from a concentration range from 0 to 50 μg/mL. Cytotoxicity test showed that a concentration of 0 to 10 μg/mL of MPLA was non-toxic to MLE-12 cells (Fig. 3A). In previous studies, we demonstrated that MPLA at a concentration of 1 μg/mL played an effective role in radiation protection [12]. Therefore, 1 μg/mL of MPLA was used in the subsequent experiments. MPLA is a TLR4-specific agonist. Previous studies using mice models have shown that MPLA protects against ionizing radiation damage to intestines by activating the TLR4 signaling pathway [11]. Interestingly, ionizing radiation modulates the expression of TLR4 in the lung tissue [12]. The data from TCGA and GTEx databases showed that the expression of TLR4 was higher in normal lung tissue than in tumor tissue (Fig. 3B). In the present study, the expression of TLR4 in MLE-12 cell, Beas-2B cell, and macrophage RAW264.7 was analyzed using the Western blot assay. We found that TLR4 expression was very low in MLE-12 and Beas-2B cells with or without MPLA treatment and radiation treatment. While TLR4 was highly expressed in RAW264.7 cells particularly after radiation, MPLA treatment did not upregulate TLR4 expression in RAW264.7 cells (Fig. 3C, D). Considering the differential expression of TLR4 in different cell types of the lung tissue, we assessed whether MPLA, a TLR4 agonist, directly enhanced the survival and alleviated irradiation-induced damage on MLE-12 cells or by activating macrophages (highly expressed TLR4). Then we subjected a clonal formation assay, as we could see, the supernatant of RAW264.7(RAWsup group) had no radiation protection effect against MLE-12, but it significantly reduces cell death after irradiation in the MPLAsup group(Fig. 3E). The “multi-target-single-hit model” showed that MPLA has no direct radioprotective effect on MLE-12 cells (Fig. 3F). Previous studies have shown that macrophage-derived exosomes in the testis are radioprotective [12]. The biological functions and pathways regulated by TLR4 were explored by functional enrichment analysis using data from the TCGA database. The GO results showed that TLR4 activity was enriched in exosome-related terms, such as secretory granule membrane (Fig. 3G). Therefore, RAW264.7 was treated with GW4869 (an inhibitor of exosome biogenesis/release) for 2 h before MPLA. Surprisingly, clonal formation assay revealed no significant radio-protection effect between the GW4869 + MPLA and the MPLAsup group (Fig. 3H, I). These results implied that MPLA protected lung tissues against irradiation damage by stimulating macrophages to produce exosomes.

### Macrophage-derived exosomes inhibited MLE-12 cell apoptosis

**Fig. 4:**
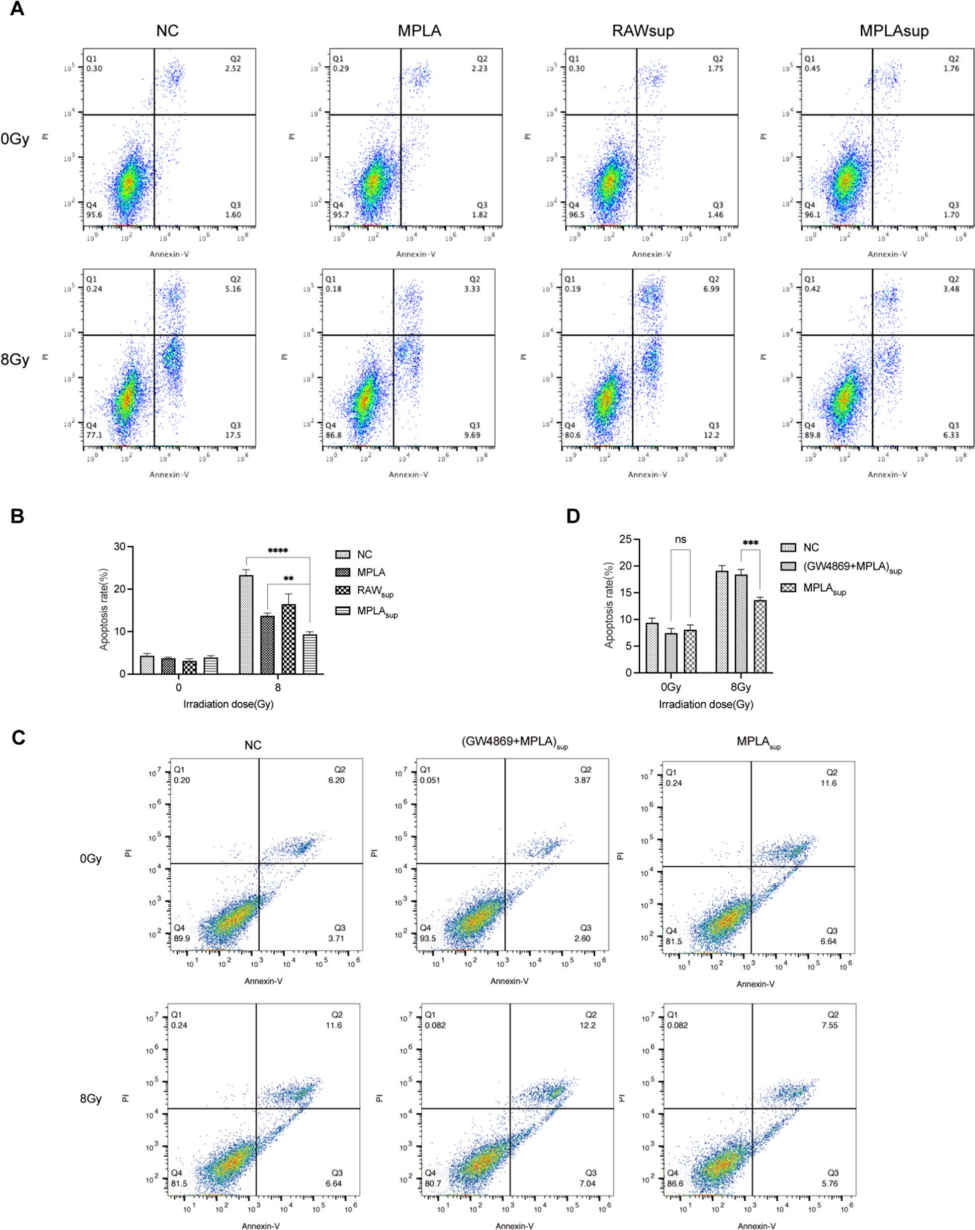
Macrophage-derived exosomes following MPLA stimulation inhibited MLE-12 cell apoptosis. **A**, **C**, Apoptosis of MLE-12 cells exposed to different IR as determined by flow cytometry analysis. **B**–**D**, The quantification of MLE-12 cells apoptosis. *P < 0.05, **P < 0.01, *** P < 0.001,****P < 0.0001

A previous study demonstrated that thoracic irradiation stimulated the apoptosis of Type II Alveolar Epithelial Cells (AECIIs) [22]. In the present study, we explored the effect of MPLA on the apoptosis of MLE-12 cells after irradiation using flow cytometry. The experimental result proves that the total number of apoptosis detected in MPLAsup group was significantly less than that in groups NC and MPLA (Fig. 4A, B). Next, we inhibited exosomes with GW4869, surprisingly, in (GW4869 + MPLA)sup group, the inhibition of apoptosis was barely observed (Fig. 4C, D). This result suggests that macrophage-derived exosomes play a key role in MPLA-mediated radioprotection.

### Macrophage-derived exosomes alleviated DNA damage in MLE-12 cells

**Fig. 5:**
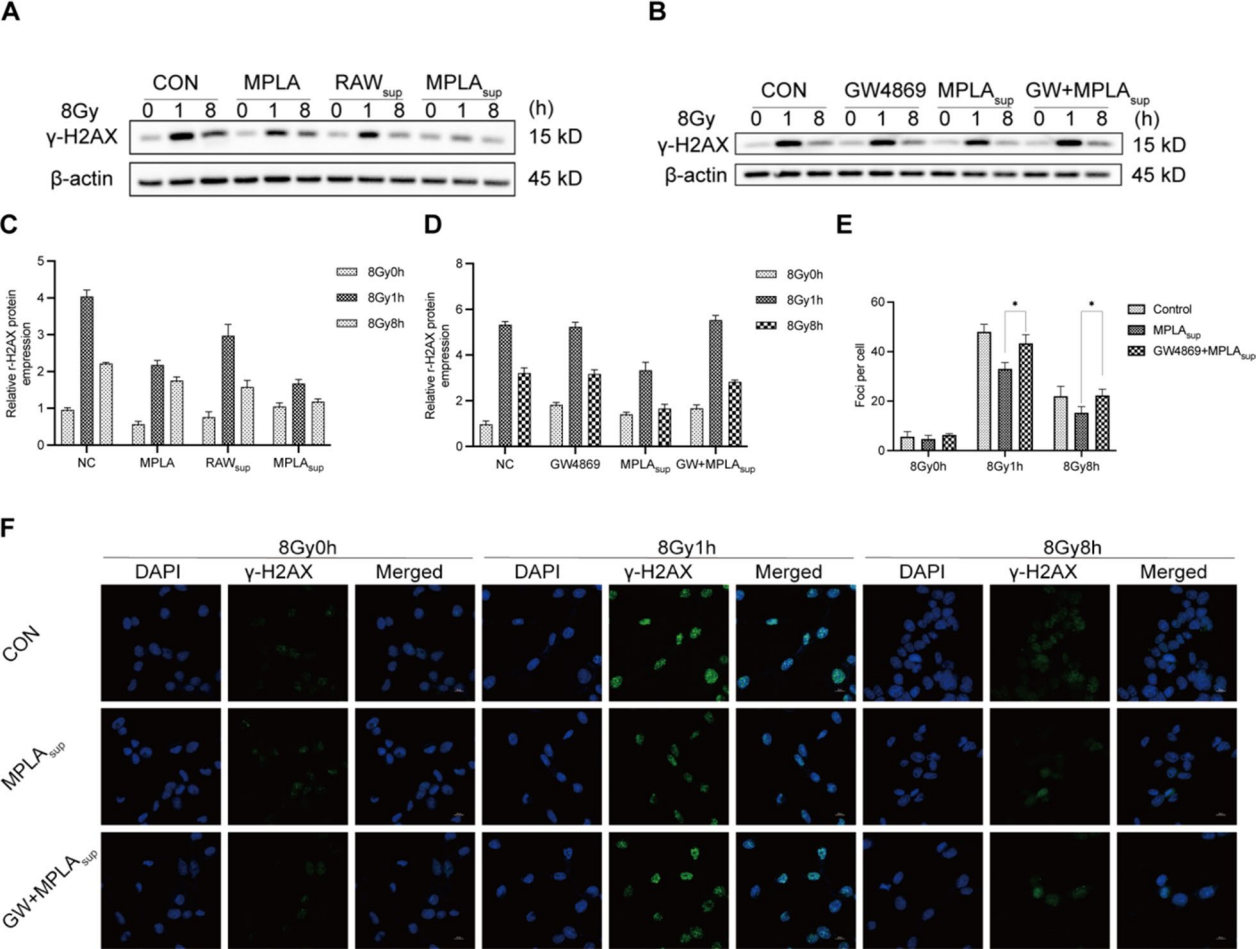
Macrophage-derived exosomes alleviated DNA damage in MLE-12 cells exposed to different irradiations. **A**, **B**, γ-H2AX expression level was determined by western blot assay. **C**, **D**, Relative qualification of γ-H2AX content in MLE-12 cells after irradiation. **E**, The number of γ-H2AX foci per cell in MLE-12 cells exposed to IR. **F**, Immunofluorescence staining of MLE-12 cells after γ-H2AX staining (green) and DAPI staining (blue). *P < 0.05, **P < 0.01, *** P < 0.001,****P < 0.0001

IR induces breakage of double-stranded DNA breaks and the subsequent apoptosis of corresponding cells. In the present study, we used γ-H2AX expression as an indicator of double-stranded DNA breaks at different time points after irradiation. The result showed that γ-H2AX overexpressed in CON group at 1 h after irradiation and partially recovered at 8 h after irradiation. γ-H2AX expression in MPLAsup group at 1 h and 8 h after radiation was significantly lower than that in the CON group (Fig. 5A, C). This experiment illuminated that MPLAsup alleviated radiation-induced DNA damage. Identically, we then used GW4869 to inhibit the exosomes from macrophage stimulated by MPLA, there was no significant difference in γ-H2AX expression between group (GW4869 + MPLA)sup and group CON, whether it was 1 h or 8 h (Fig. 5B, D). Then, we detected the γ-H2AX foci by immunofluorescence method, numbers of γ-H2AX foci were much higher in IR group compared to MPLAsup group, the (GW + MPLA)sup group had no significant difference to the IR group (Fig. 5E, F). Overall, the results showed that macrophage-derived exosomes alleviated DNA damage in MLE-12 cells.

### Exosomes derived from classically activated M1 macrophage suppress EMT

**Fig. 6:**
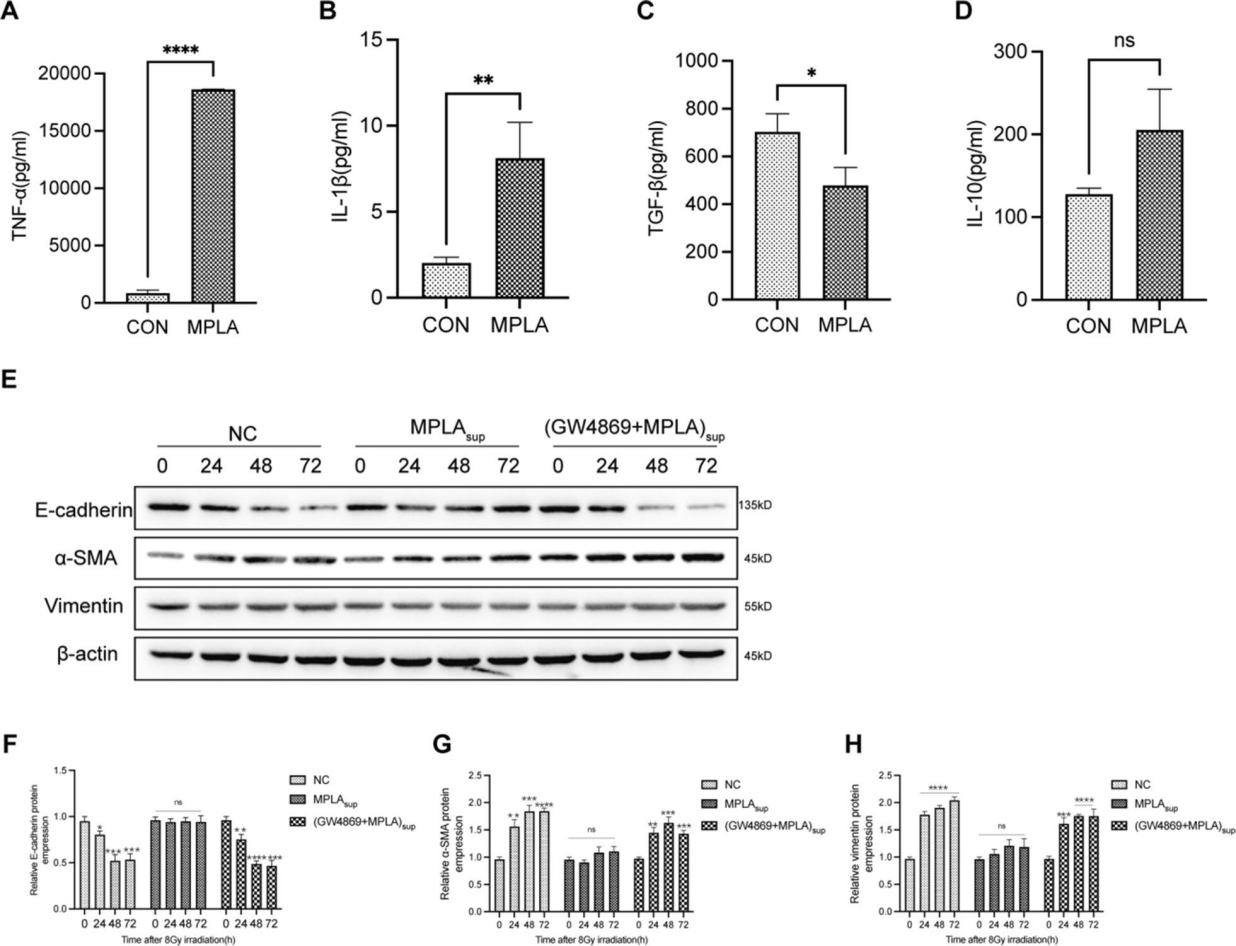
M1-type macrophage-derived exosomes reversed EMT. **A**–**D**, The levels of TNF-α (**A**), IL-1β (**B**), TGF-β (**C**), and IL-10 (**D**) in the supernatant secreted by RAW 264.7 cells treated with or without MPLA. **E**, The expression of E-cadherin, α-SMA and vimentin were determined in MLE-12 cells by western blot assay. **F**–**H**, Relative qualification of E-cadherin, α-SMA and vimentin expression level in MLE-12 cells after irradiation. Ns means no significantly different. *P < 0.05 vs CON group or 0 h group. **P < 0.01 vs CON group or 0 h group, *** P < 0.001 vs CON group or 0 h group, ****P < 0.0001 vs CON group or 0 h group

Classically activated M1 macrophages promote inflammation and inhibit fibrosis, while alternatively activated M2 macrophages modulate inflammation and promote fibrosis [23, 24]. Lipopolysaccharide (LPS) induces M1 polarization of macrophages [25, 26]. To clarify the effect of MPLA on macrophage polarization, RAW264.7 cells were cultured in media supplemented with MPLA. The supernatant was collected after 12 h of culture to analyze the expression levels of TNF-α, TGF-β, IL-1β, and IL-10. We found that TNF-α and IL-1β were over secreted, suggesting that MPLA stimulated M2 polarization of RAW264.7 cells (Fig. 6A–D).

Irradiation induces EMT of epithelial cells following pulmonary fibrosis [27]. Our results showed that the expression of the epithelial marker E-cadherin was decreased and the expression of the mesenchymal markers Vimentin and α-SMA was upregulated after irradiation. However, the overexpression of α-SMA and Vimentin was inhibited in the MPLAsup group. Meantime, the downregulation of epithelial markers E-cadherin was inhibited in the MPLAsup group. There were no differences in the GW + MPLAsup group compared with the IR group(Fig. 6E–H). In conclusion, our experiments demonstrated that macrophage-derived exosomes can attenuate RILI. The radiation protection effect might be related to inhibition of EMT process.

## Discussion

**Fig. 7:**
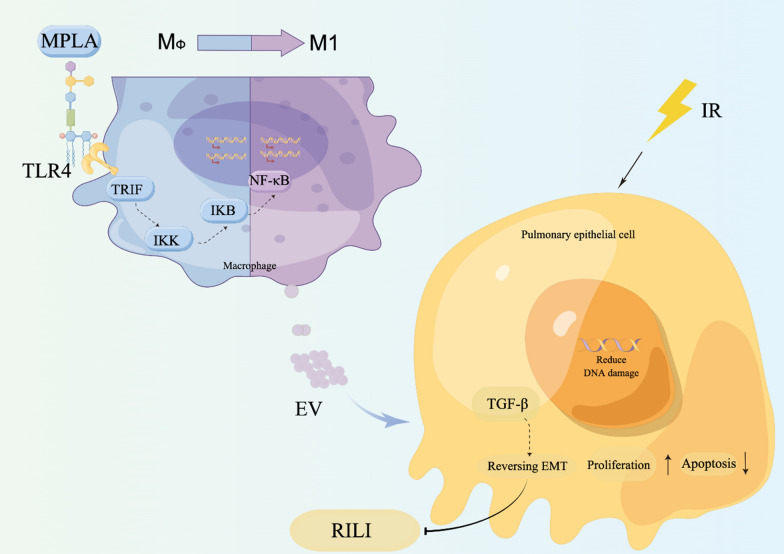
Potential mechanism of MPLA exerts radioprotective effect on RILI. (Figure By Figdraw)

This study showed that MPLA inhibited or ameliorated RILI by stimulating the production of exosomes by macrophages. The research demonstrated that MPLA could reduce lung tissue damage after irradiation, suggesting that MPLA had a specific inhibitory effect on early pneumonia of RILI. Furthermore, the apoptosis of lung epithelial cells was significantly reduced after irradiation. Next, we found that MPLA attenuated radiation-induced lung fibrosis at 16-week post-radiation. Moreover, the results showed that MPLA reversed lung epithelial cell EMT. After confirming the radiation protective effect of MPLA in vivo, we tried to explore the radiation protective mechanism of MPLA in vitro. Considering the low expression of TLR4 in normal lung cells and high expression of TLR4 in macrophages, the radiation protection effect of MPLA might be related to macrophage-derived exosomes. Then we used clonogenic assays to demonstrate that macrophage-derived exosomes promote the proliferation of MLE-12 cells after irradiation. These results interested us so that we did the futher assays. Then we illuminated that MPLA stimulated macrophages to M1-type polarization, secretes exosomes and ameliorates RILI.The potential mechanism of MPLA in RILI is shown in Fig. 7(By Figdraw).To sum up, MPLA is a novel drug that protect against RILI.

Ionizing radiation induces the differentiation of lung fibroblasts to differentiate into myofibroblasts [28–30]. Myofibroblasts cause excessive deposition of extracellular matrix and abnormal remodeling, leading to RILI by promoting collagen synthesis [31]. TLRs mediate resistance to irradiation in multiple animal tissues [32], including testicular tissue, intestinal epithelium, and other tissues and organs [11, 12]. TLR4 also protects against RILI. A previous study showed that both TLR2 and TLR4 enhance radiation-induced fibrosis in mice [33]. Another study showed that HKST, an agonist of TLR2, TLR4, and TLR5, ameliorated RILI [14]. However, the expression of TLR4 varies between different human tissues, which explains the variation in radioprotective effects. TLR4 is underexpressed in the testis tissues and highly expressed in the spleen tissues. Macrophage-derived exosomes exert radioprotective effects on the testis [12], suggesting that TLRs could alleviate RILI. The mechanism by which TLRs alleviate radiation-induced damage is unclear and needs further exploration. In this study, we found that MPLA protects against IR damage to lung tissue. However, TLR4 expression in lung tissue and lung alveolar epithelial cells is very low. We demonstrated that MPLA reduces radiation-induced cell apoptosis and promotes the proliferation of lung epithelial cells by stimulating the secretion of exosomes in macrophage. Meanwhile, analysis of data in the TCGA revealed that TLR4 expression was significantly lower in lung tumor tissue in normal lung tissue.

Recent studies have shown that EMT plays an essential role in the development of radiation-induced pulmonary fibrosis [13]. Research has shown that reversing EMT can reduce RILI. For instance, Re-Du-Ning ameliorated radiation pneumonitis by inhibiting EMT [34]. ECM-derived hydrogels prevent RILI by modulating EMT [35]. Polydatin alleviates RILI by inhibiting EMT [36]. Herein, an in vitro experiment revealed that MPLA promotes the polarization of RAW 264.7 cells towards M1 phenotype. Studies have demonstrated that M1 macrophages promote inflammation and inhibits fibrosis [23]. Interestingly, LPS treatment (induces macrophage polarization to M1 type) attenuates early radiation damage to the lung [37]. Radiation-induced early inflammation plays a minimal role in the later development of pneumonia [38]. TGF-β1 is a specific cytokine that induces extracellular matrix production [39, 40], consistent with our findings. TGF-β promote radiation-induced EMT process [41, 42] Nevertheless, the relationship between TLRs and EMT is not well understood. This study revealed that MPLA promotes the polarization of RAW 264.7 cells towards the M1 phenotype and inhibits TGF-β secretion by macrophages. Moreover, the macrophage-derived exosomes inhibited the radiation-induced EMT.

RILI encompasses any lung toxicity induced by radiation therapy andmanifests acutely as radiation pneumonitis and chronically as radiation pulmonary fibrosis. It is believed that the main mechanism is that when the lung tissue is under radiation, a large amount of reactive oxygen species can be produced, and act on the alveolar epithelial cells and vascular endothelial cells, causing a large number of cells to undergo apoptosis and damage the barrier function of the lung tissue [1, 3]. Therefore, a lot of research has been carried out using natural antioxidants to perevent RILI [43, 44]. Mitochondria, as an important site of ROS and energy metabolism, may play a key role in the prevention of RILI [45]. In addition, exosomes are often used as therapeutic drug carriers in clinical applications [46, 47], with the rise of nanomaterials and the perfection of the principles of nanoparticle design, nanoantioxidants may be able to exert radiation protection by targeting lung tissue specifically [48, 49], which we could combine the purified TLR4-activated macrophage-derived exosomes with nanomaterials for precise treatment of RILI. On the other hand, related studies have shown that statins have significant radioprotective effects, but the mechanism remains unclear. Related studies illuminated macrophages could reduce obesity related inflammation [50, 51]. Therefore, we speculate that the radioprotective effect of macrophages may be related to fat metabolism, which is also one of our next research directions.

In conclusion, MPLA significantly alleviates radiation-induced lung injury by promoting the polarization of macrophages towards the M1 type. The M1 macrophages secrete exosomes that attenuate radiation-induced DNA damage and modulate the secretion of TGF-β that reverses radiation-induced EMT and lung fibrosis. This study demonstrates a novel mechanism for inhibiting or reversing RILI. The findings of this study provide a new perspective for protecting against IR-induced lung damages, either during treatment or accidentally.

## Data Availability

The datasets are available under reasonable request.
